# Developing a Lung Model in the Age of COVID-19: A Digital Image Correlation and Inverse Finite Element Analysis Framework

**DOI:** 10.3389/fbioe.2021.684778

**Published:** 2021-10-26

**Authors:** Mohammad Maghsoudi-Ganjeh, Crystal A. Mariano, Samaneh Sattari, Hari Arora, Mona Eskandari

**Affiliations:** ^1^ Department of Mechanical Engineering, University of California, Riverside, Riverside, CA, United States; ^2^ Faculty of Science and Engineering, Swansea University, Swansea, United Kingdom; ^3^ BREATHE Center, School of Medicine, University of California, Riverside, Riverside, CA, United States; ^4^ Department of Bioengineering, University of California, Riverside, Riverside, CA, United States

**Keywords:** lung mechanics, inverse finite element analysis, digital image correlation (DIC), heterogeneity, anisotropy, hyperelasticity, *in-silico* ventilation

## Abstract

Pulmonary diseases, driven by pollution, industrial farming, vaping, and the infamous COVID-19 pandemic, lead morbidity and mortality rates worldwide. Computational biomechanical models can enhance predictive capabilities to understand fundamental lung physiology; however, such investigations are hindered by the lung’s complex and hierarchical structure, and the lack of mechanical experiments linking the load-bearing organ-level response to local behaviors. In this study we address these impedances by introducing a novel reduced-order surface model of the lung, combining the response of the intricate bronchial network, parenchymal tissue, and visceral pleura. The inverse finite element analysis (IFEA) framework is developed using 3-D digital image correlation (DIC) from experimentally measured non-contact strains and displacements from an ex-vivo porcine lung specimen for the first time. A custom-designed inflation device is employed to uniquely correlate the multiscale classical pressure-volume bulk breathing measures to local-level deformation topologies and principal expansion directions. Optimal material parameters are found by minimizing the error between experimental and simulation-based lung surface displacement values, using both classes of gradient-based and gradient-free optimization algorithms and by developing an adjoint formulation for efficiency. The heterogeneous and anisotropic characteristics of pulmonary breathing are represented using various hyperelastic continuum formulations to divulge compound material parameters and evaluate the best performing model. While accounting for tissue anisotropy with fibers assumed along medial-lateral direction did not benefit model calibration, allowing for regional material heterogeneity enabled accurate reconstruction of lung deformations when compared to the homogeneous model. The proof-of-concept framework established here can be readily applied to investigate the impact of assorted organ-level ventilation strategies on local pulmonary force and strain distributions, and to further explore how diseased states may alter the load-bearing material behavior of the lung. In the age of a respiratory pandemic, advancing our understanding of lung biomechanics is more pressing than ever before.

## Introduction

Respiratory diseases and disorders, such as asthma, emphysema, bronchitis, pulmonary fibrosis, and lung cancer, collectively lead as the global cause of morbidity and mortality (Centers for Disease Control and Prevention, 2015; Eskandari et al., 2018). These pulmonary illnesses impose strenuous social and economic burdens, as seen with the recent lung-damaging COVID-19 outbreak (Atkeson, 2020). The acute and progressive pathological inflammation and bronchoconstriction of the lung obstruct and restrict airflow and oxygenation, inducing altered mechanical properties (Suki and Bates, 2011; Eskandari et al., 2016). This mechanical remodeling is multiscale, spanning the destruction of alveolar sac elasticity in emphysema (Suki et al., 2003), the over stiffening of the parenchymal tissue in pulmonary fibrosis (Faffe and Zin, 2009), and the constriction and collapse of airways in asthma (Bai and Knight, 2005; Eskandari et al., 2015; Maghsoudi-Ganjeh et al., 2021). Thus, the hierarchical and complex structure of the lung highlights the importance of mechanics in respiratory health (Tawhai and Bates, 2011; Eskandari et al., 2013).

Despite the growing body of literature on pulmonary mechanics, the multiscale and multiphysics link between the global pressure-volume behavior of the lung and the local-level tissue deformation remains largely unexplored. There has been notable progress to characterize the lung at the organ scale through classical pressure-volume curves and at the tissue level using indentation and uniaxial tensile tests (Lai-Fook et al., 1976; Zeng et al., 1987; Fung, 1988; Eskandari et al., 2018); however these investigations remain siloed at disconnected scales. Amalgamating these multiphysics and multiscale behaviors is central to understanding lung disease mechanisms, predicting disease progression, and mitigating ventilator-induced-lung-injuries (VILI) to eliminate tissue over stretching (volutrauma) and stressing (barotrauma) (Dreyfuss and Saumon, 1998; Vlahakis et al., 1999; Arora et al., 2017; Arora et al., 2021). Unless an atlas for pulmonary kinetics and kinematics can be established, current ventilation protocols will continue to be subject to trial and error approaches and hindered from advancements despite exigent demands instilled by a worldwide pandemic (The Acute Respiratory Distress Syndrome Network, 2000; Amato et al., 2015).

Advancements in biologically-oriented digital image correlation (DIC) techniques have facilitated quantifying the mechanical connections between organ-level breathing and local tissue behavior for fast, large, and non-linear deformations. DIC is a common full-field, non-contact deformation characterization technique originally applied on inert structures (Chu et al., 1985), and has now been enhanced to study the behavior of intricate biological tissues, such as the cornea (Boyce et al., 2008), arteries (Sutton et al., 2008), knees (Mallett and Arruda, 2017), and most recently, the lung (Mariano et al., 2020). In this method, sequential images of a specimen’s speckled surface undergoing loading are used to obtain the topological displacement field (Chu et al., 1985). While DIC describes the kinematics, inverse finite element analysis (IFEA) can be employed to divulge the kinetics. IFEA yields specimen mechanical properties by minimizing the error between the displacements predicted by the Finite Element (FE) model and those measured via experiment (Birzle et al., 2019).

Here we construct the first in-silico IFEA structural representation of the whole lung as informed and validated from DIC resulting from applied evolutionary pressure-volume loading controlled by a custom-designed breathing apparatus (Mariano et al., 2020; Sattari et al., 2020). Based on the obtained surface geometry and deformation map of the inflating lung, a corresponding reduced-order 3-D FE model is constructed using membrane elements undergoing the same experimental lung pressures. Various constitutive models are explored, including homogeneous isotropic hyperelastic, homogeneous anisotropic hyperelastic, and heterogeneous isotropic linear-elastic materials (Mooney, 1940; Holzapfel et al., 2000). The parameters of the multiple FE models are calibrated through a fully automated IFEA framework. Both classes of derivative-based and gradient-free optimization algorithms are implemented to predict the material response by minimizing the error between model-predicted and DIC-recorded displacements of the external surface of the lung. The set of calibrated material parameters, along with local and heterogeneous deformation results of the in-silico lung, are presented, model performances are compared, and future applications are discussed.

## Materials and Methods

### Digital Image Correlation and Pressure-Volume Experiments

Previously established extensive experimental DIC protocols and pressure-volume tests were utilized for the *ex-vivo* specimen tests conducted here and will be briefly summarized (Mariano et al., 2020; Sattari et al., 2020). Fresh porcine lungs from an abattoir were obtained (50 kg female domestic York farm minipigs, Institutional Animal Care and Use Committee approval not required) and a plastic tube was inserted through the trachea to fully inflate the lung using an airline pressure system. A generic exfoliator pad dipped in quick-drying white enamel paint (rust-oleum) was used to create speckles (Mariano et al., 2020). The specimen was loaded into our custom pressure-volume apparatus for controlled inflation tests; this device consisted of two pistons (a source and a response), a transparent tank, and a computerized controller system (Sattari et al., 2020). 900 ml of air was applied to the lung, and the real-time continuous pressure and actual volumetric deformation of the lung (less than the applied volume due to the compression of air) was measured. As in previous studies, a preload of 5 cmH2O was used as the reference state. A rate of 15 breaths-per-minute was used and the specimen was preconditioned three times to generate reproducible cycles and the fourth inflation response was analyzed.

**FIGURE 1:**
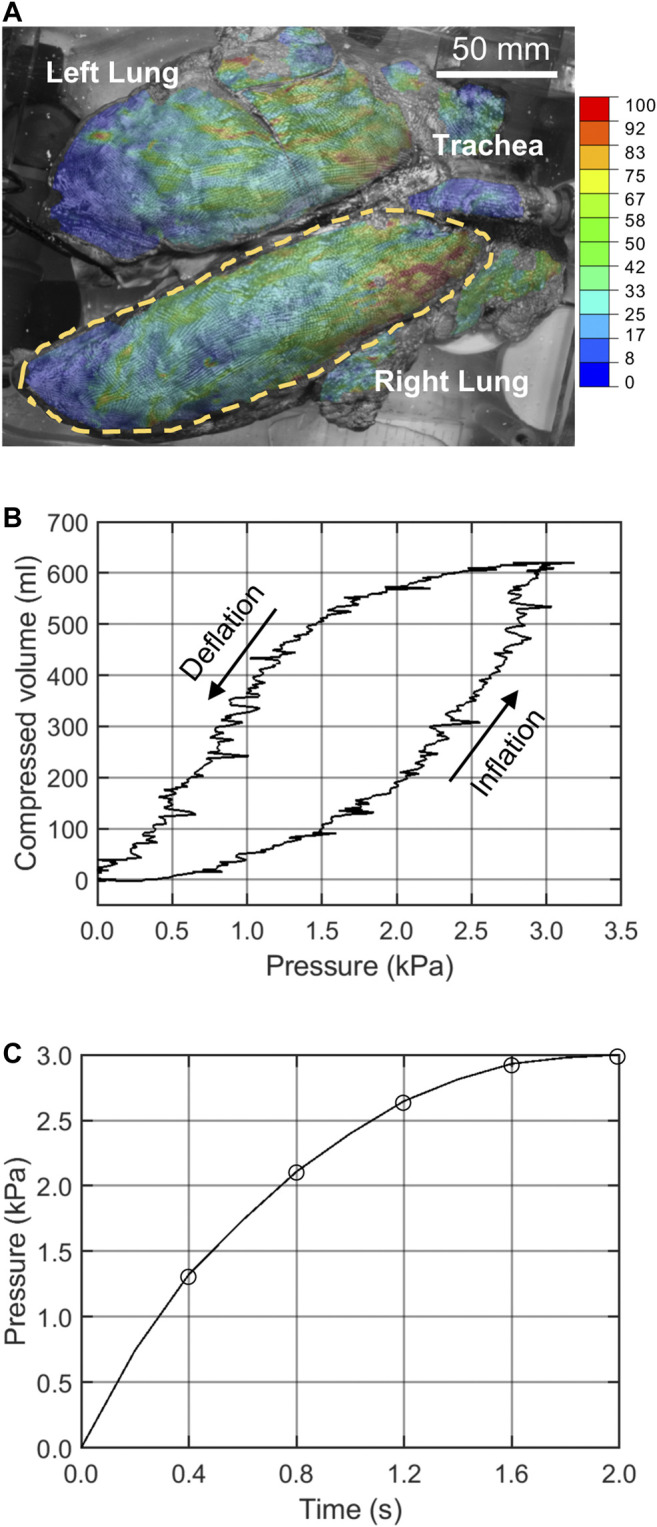
**(A)** The right lung lobe was selected for analysis and DIC strains are shown. **(B)** The pressure-volume data of the stabilized inflation cycle was used to extract the **(C)** pressure-time data applied to the FE model.

The 3-D stereoscopic DIC system (ARAMIS 12M, Trilion Quality Systems) consisted of two optical cameras hovering over the transparent tank, which recorded the dynamic deformation of the lung at 10 Hz. For a measuring volume of 375 × 295 × 295 mm, the calculated displacement measurement accuracy was 0.10875 mm (Jones and Iadicola, 2018). The images were analyzed following standard DIC techniques to calculate the displacement and strain of the exterior surface of the lung relative to its uninflated state (Chu et al., 1985; Jones and Iadicola, 2018) and are extensively detailed in Mariano et al., 2020. Figure 1A showed the DIC strain map corresponding to the peak pressure-volume inflation stage. Figure 1B showed the corresponding pressure-volume curve obtained from the inflation. Based on this curve and the inflation rate, the pressure-time amplitude curve (Figure 1C) was extracted and applied to the FE model as the loading step.

### Inverse Finite Element Analysis Overview

**FIGURE 2:**
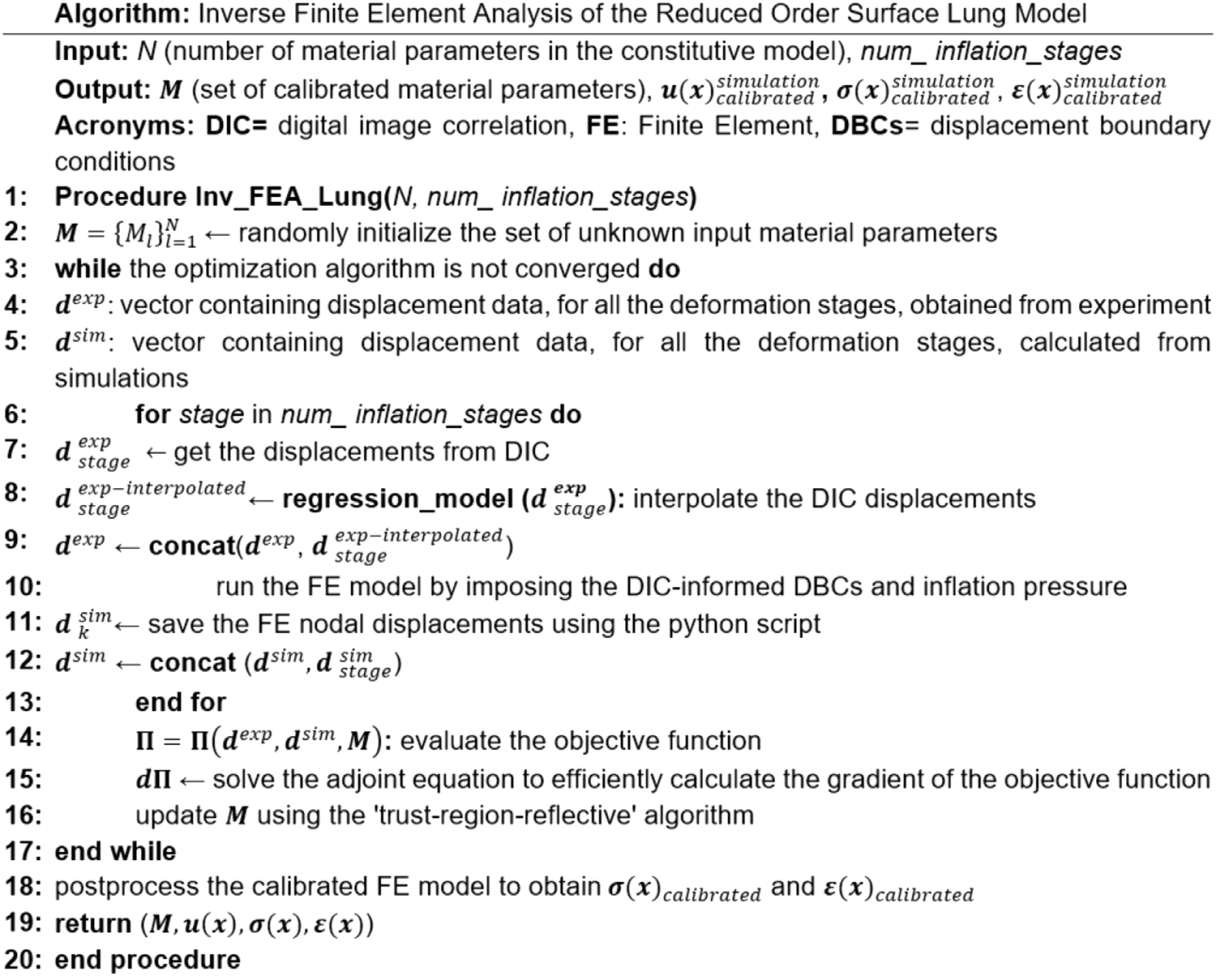
The overall algorithm for the IFEA framework implementation. The model started with an initial estimate for the material parameters, then the optimization algorithm incrementally moved forward toward the optimal solution by minimizing the displacement error between model predictions and DIC measurements. In this workflow, u refers to the nodal displacement, σ is the Cauchy stress, ε is the technical strain.

The FE models were calibrated using the measured DIC displacement of the *ex-vivo* lung. The algorithm (shown in Figure 2) minimized the error by finding the optimal values for unknown free material parameters. The inverse FE model leveraged several known parameters: the surface geometry of the lung from the uninflated stage obtained after three preconditioning inflation-deflation cycles, the experimentally measured pressure-time graph, and the DIC displacement field of the lung surface. However, the type of constitutive model and corresponding material parameters were unknown; these model attributes were left to be determined by the optimization algorithms. Before applying the model to the actual lung, we verified that when applied on a simpler geometry with known deformation field and material properties the model is able to recover the given material parameters successfully.

Various constitutive relations were examined. The material parameters were initialized from several distinct starting points and the solution was generated multiple times to ensure mathematical robustness. The error was calculated based on the normalized squared sum of residuals. Material properties were perturbed to calculate the sensitivity of the error, which informed the alternate directions adopted by the optimization algorithm. This incremental procedure was repeated to progressively minimize the error until a pre-defined convergence criterion was met (change in the error less than 10^−6^ of the initial value). Given the nonlinear lung pressure profile (Figure 1C), the displacement error was evaluated at several increments evenly spaced out throughout the inflation cycle and not just at the full inflation point.

### Finite Element Model Organization

The built-in stereo camera DIC system was used to capture the exterior surface of the lung. The obtained geometry was tessellated with 3-D triangular elements averaging 1 mm in size. The DIC system could only detect and analyze the visible portion of the lung lobes (Figure 1). The raw data from the original DIC geometry was not suitable for the FE simulation as it contained some elements with poor isoperimetric quality and sharp surface discontinuities. To improve the mesh quality, MeshLab (ISTI-CNR, Italy) was used to smoothen the surface while preserving the original surface features (Cignoni et al., 2008). Two fine and coarse meshed models, with ∼5,000 and 457 elements respectively, were exported as STL files which were then converted to Abaqus input files using the built-in script plugin (Dassault Systems, Providence, RI, United States). The fine and coarse meshes were used to study the cases corresponding to homogeneous and heterogeneous material models, respectively. This approach reduced the number of unknown material parameters in the heterogeneous case and was necessary to substantially decrease the IFEA computational cost. After confirming the mesh resolution was sufficient, both meshed models were discretized using 3-D membrane elements (M3D3). The recorded DIC experimental displacement values of the nodes sitting on the perimeter of the surface geometry were applied as displacement boundary conditions to the FE models; as such, while the geometry of the model represented the visible portions of the lung lobes, the role of the adjacent tissue was represented through the application of these periphery nodal displacements (as opposed to traditional boundary conditions). The thickness of membrane elements were set to 1.0 mm.

One-to-one experimental to FE model nodal correspondence was created for error calculations by probing the displacement from ∼7,000 evenly distributed points across the surface. During the multiple inflation stages, where the displacement error between the FE model and DIC nodes were to be calculated, there is not a one-to-one correspondence between FE model nodes and DIC probe points. Therefore, interpolation must be used to find FE model nodes corresponding to the DIC probe points so that the error could be computed. An interpolation technique was used, utilizing the DIC displacements and coordinates of the probe points to train the *k*-nearest-neighbor algorithm (Altman, 1992). The performance of the interpolation model was evaluated using 10-fold cross validation, confirming the accuracy was above 0.95. The number of nearest neighbors *k* was set to be five. The interpolation technique was implemented using two Python scripts: one to access nodal coordinates from Abaqus, and one to perform interpolation.

The FE model was solved using Abaqus dynamic implicit solution scheme by subjecting the model to the experimental lung pressure values at five deformation stages (Figure 1C). Nonlinear geometry formulation was utilized, and the displacement and strains were analyzed. In order to significantly accelerate the IFEA process, all the scripts were parallel-coded such that multiple FE simulations were running simultaneously in batch mode.

### Constitutive Models

The best performing constitutive model was not known *a priori.* We investigated three different material model cases to consider homogeneity versus regional heterogeneity, preferential orientation using an anisotropic versus isotropic response, and the linear versus nonlinear cases to determine optimal constitutive parameters as detailed below. It was important to note the reduced-order nature of the model meant the parameters of these constitutive models were not simply the material properties of the lung; rather they are a pseudo-material model, represented as a projected, averaged surface response of tissue or compound material parameters of the parenchyma, airways, and pleura layer consolidated together.

#### Homogeneous Isotropic Hyperelastic Case

Here the compressible Mooney-Rivlin hyperelastic model (Mooney, 1940) with the strain energy density defined as $W=C_{10}\left({\bar{I}}_{1}-3\right)+C_{01}\left({\bar{I}}_{2}-3\right)+\frac{1}{D_{1}}{\left(J-1\right)}^{2}$ was used. ${\bar{I}}_{1}$ , ${\bar{I}}_{2}$ were the first and second invariants of the deviatoric deformation tensor, J was the Jacobian of deformation gradient F , and $C_{10},C_{01\;},$ and $D_{1}$ were the three unknown material parameters. The stress-stretch curve of this model can span strain-hardening and strain-softening behaviors, depending on the relative values of the material parameters, allowing a versatile IFEA framework.

#### Homogenous Anisotropic Hyperelastic Case

The Holzapfel-Gasser-Ogden (HGO) formulation (Holzapfel et al., 2000) with the strain energy density defined as $W=W_{iso}+W_{aniso}$ was used here. $W_{iso}$ and $W_{aniso}$ were defined as $W_{iso}$ = $C_{10}\left({\bar{I}}_{1}-3\right)+\frac{1}{D}\left(\frac{J^{2}-1}{2}-\ln J\right)$ and $W_{aniso}=\frac{k_{1}}{2k_{2}}\left[exp\left\{k_{2}{\left[k\left({\bar{I}}_{1}-3\right)+\left(1-3k\right)\left({\bar{I}}_{4}-1\right)\right]}^{2}\right\}-1\right]$ . The five unknown material parameters were $C_{10},D,k_{1},k_{2}$ and k . The three strain-representing kinematic variables were ${\bar{I}}_{1}$ , ${\bar{I}}_{4}$ , and J . In this formulation ${\bar{I}}_{1}$ was the first invariant of the deviatoric deformation tensor. In addition, ${\bar{I}}_{4}$ was the pseudo-invariant of $\bar{C}$ and $a_{0}⊗a_{0}$ where $\bar{C}=J^{-2/3}C$ followed the multiplicative decomposition of the deformation gradient F and the deformation tensor $C=F^{T}F$ . The vector $a_{0}$ was a unit vector field defining the fiber direction in the undeformed configuration. J was the Jacobian of deformation gradient F . ${\bar{I}}_{4}$ represented the squared of the stretch ratio of the material fiber λ in the direction of the fiber family defined by $a_{0}$ . The degree of preferential alignment of the fiber family governing anisotropy was controlled by the dispersion parameter k∈[0,1/3] , where 0 indicated the family of fibers were fully aligned and 1/3 indicated a completely random distribution of the fiber family (reducing to isotropic form). For each element in the mesh, the local *z*-direction was the outward normal to the element surface, the local *y*-direction was specified in the anterior-posterior direction, and the local *x*-direction was subsequently determined based on the right-hand sign convention for Cartesian coordinates. Given that strains were smaller in the medial-lateral direction, $a_{0}$ was aligned with the defined local *x*-direction for each element. It should be noted that even though we define *x*-axis for the main fiber directions, since the parameter k is left free to be determined by the optimization engine, the true fiber directions are not strictly fixed in the model.

#### Heterogeneous Isotropic Linear-Elastic Case

In this case we considered a linear elastic isotropic material model (Eskandari and Kuhl, 2015) with Young’s modulus E and Poisson’s ratio υ . The regional heterogeneity of this model meant each element of the mesh had its own two parameters that were found by the optimization algorithm. The shear modulus μ of each element was then calculated using $\mu =\frac{E}{2\left(1+\nu \right)}$ and regionally mapped onto the lung surface.

### Optimization Algorithms for Extracting Material Parameters

Gradient-based optimization algorithms are prone to returning local optima in the neighborhood of the initial search point, while the derivative-free optimization algorithms, such as meta-heuristic algorithms, are more likely to return the global optimal solution instead (Nocedal and Stephen, 2006). Therefore, two broad classes of gradient-based and gradient-free optimization algorithms were implemented to improve global optimum acquisition. In the gradient-based approach, the trust-region-reflective (TRR) algorithm (Steihaug, 1983), available in Matlab *lsqnonlin* function (The MathWorks Inc., MA, United States), was used for the two homo/iso/hyper and homo/aniso/hyper cases; and the sequential-quadratic-programming (SQP) algorithm (Boggs and Tolle, 1995), available in Matlab *fmincon* function, was used for the hetero/iso/linear-elastic case. As for the gradient-free approach, we implemented our own version of the particle swarm optimization (PSO) algorithm (Kennedy and Eberhart, 1995) in a Matlab script. This provided more flexibility to impose custom constraints on our problem, such as bounds on the position and velocity of particles, which may not be done so freely in the built-in Matlab PSO algorithm. The IFEA processes in both approaches were fully automated by conjoining Matlab, Abaqus, and Python. To help avoid local optima, the TRR and SQP algorithms were run from several randomly selected initial estimate points within the search space as given in Table 1 and Table 2. As a verification step, we applied the IFEA framework to a test case model with a simpler geometry and known deformation field and material parameters. We confirmed that the optimization pipeline was indeed successful in recovering the pre-known material parameters.

**TABLE 1:** Sets of initial estimates and converged optimal material parameters for the homo/iso/hyper case. In order to avoid local minimum, the optimization routine was repeated from seven different starting points.

	Initial material parameters			Optimal material parameters		
**Run #**	$C_{10}\;$ **(kPa) [1–200]**	$C_{01}\;$ **(kPa) [1–200]**	$D_{1}\left(\times 10^{-4}\right)\;kPa^{-1}$ **[10^−4^–10^−2^]**	$C_{10}$ **(kPa)**	$C_{01}$ **(kPa)**	$D_{1}$ $\left(\times 10^{-4}\right)\;kPa^{-1}$
1	25	10	5	136.2	1.0	13.2
2	10	20	15	136.6	1.0	13.5
3	100	42	61	136.6	1.0	13.5
4	172	18	32	136.9	1.0	13.6
5	10	90	80	136.1	1.0	13.2
6	50	50	50	136.7	1.0	13.6
7	200	150	90	136.5	1.0	13.4

**TABLE 2:** Sets of initial estimates and converged optimal material parameters for the homo/aniso/hyper case. In order to avoid local minimum, the optimization routine was repeated from seven different starting points.

	Initial material parameters					Optimal material parameters				
**Run #**	$C_{10}\;$ **(kPa) [1–200]**	D **(×10^−4^)** $\;kPa^{-1}$ **[10^−4^–10^−2^]**	$K_{1}$ **(kPa) [1–200]**	$K_{2}$ **[0–1]**	κ **[0–0.33]**	$C_{10}$	D **(×10^−4^)**	$K_{1}$	$K_{2}$	κ
1	18	42	8	0.30	0.11	116.5	42	1.0	0.05	0.33
2	51	28	14	0.70	0.05	116.6	28	1.0	0.24	0.33
3	80	12	100	0.45	0.2	116.2	12	1.0	0.09	0.33
4	150	10	76	0.62	0.3	116.0	10	1.0	0.13	0.33
5	7	100	35	0.90	0.17	116.3	100	1.0	0.15	0.33
6	180	63	22	0.10	0.17	116.5	63	1.0	0.07	0.33
7	100	50	150	0.18	0.25	116.5	50	1.0	0.13	0.33

#### The Adjoint Method to Calculate the Objective Functional Gradient

In this study, the adjoint method was used to calculate the gradient for the hetero/iso/linear-elastic case given that the total number of unknown material parameters for this case was much greater than that of the homo/iso/hyper (three parameters) and homo/aniso/hyper (five parameters) cases; In the former case there were 457 elements and two material unknowns at each element, which rendered the total number of unknown parameters to 914. If the classical objective function gradient with finite difference methods were used, each iteration of the optimization algorithm for the hetero/iso/linear-elastic forward elasticity problem would have to be solved 914 × 2 = 1828 times based on the central difference method to approximate the sensitivity of the objective function. As such, the optimization algorithm would be rendered prohibitively expensive and therefore, adjoint methods for optimization were utilized (Oberai et al., 2003). This effective method required solving the problem only twice; once for the forward elasticity problem and once for the adjoint problem.

The derivation of the adjoint method to formulate our IFEA problem employed the objective functional below, (with the predefined measure of error between simulation and experiment):

Π=Π(u,p), (1)

where Π was the objective functional, vector u was the global displacement vector, and p was the set of unknown material parameters. Note that u was dependent on the p because knowing the material parameters allowed us to run the forward elasticity problem and solve for the nodal displacements. Therefore, the dependence of Π to p was implicit. The size of the vectors **u** and **p** were u:M×1 and p:N×1 , where M was the total degrees of the freedom of the FE model. Specifically, our mesh had 263 nodes where each node has three translational degrees of freedom (no rotational degrees), and therefore, M=263×3=789 . The size of vector p for 457 meshed elements with two unknown material parameters E and ν was N=2×457=914 . Since Π represented a measure of error, it took the following form:

$$\Pi =\frac{1}{2}\times \sum_{n=1}^{n_{\mathrm{max loading}}}\|\left(u_{n}^{\mathrm{sim}}-u_{n}^{\exp}\right)\|+\rho \left(p\right),$$ (2)

where the first term was the L-2 norm of the error, and the second term as a regularization parameter to tackle the ill-posed aspect of the inverse problem (Isakov, 2017). One popular choice for ρ could be $\rho =\frac{\alpha }{2}p$ , where α was a regularization parameter selected to be a very small number (10^−6^) selected based on the theory of residues (Tikhonov et al., 2013). The regularization was only applied to the heterogeneous model with the gradient-based optimization. In our case, n_maxloading referred to the five time points at which the error was calculated through the full inflation path.

The derivative of Π for the optimization algorithm was defined as:

$$d\Pi ={\left(\frac{\partial \Pi }{\partial p}\right)}^{T}\mathrm{dp}+{\left(\frac{\partial \Pi }{\partial u}\right)}^{T}\left(\frac{\partial u}{\partial p}\right).$$ (3)

The values for $\frac{\partial \Pi }{\partial p}$ and $\frac{\partial \Pi }{\partial u}$ were known given their explicit definition in Eq. 1. In order to get the term $\frac{\partial u}{\partial p}$ , the forward elasticity problem had to be solved since in general we do not have an analytical relation between u and p . The forward elasticity problem was cast into the following standard discretized format obtained from the FE model:

K(p)u=f(p), (4)

where K:M×M was the global stiffness matrix, and f:M×1 was the global load vector. From there the partial derivative of Eq. 4 was:

$$\left(\frac{\partial K}{\partial p}\right)u+K\left(p\right)\left(\frac{\partial u}{\partial p}\right)=\frac{\partial f}{\partial p},$$ (5)

where

$$\frac{\partial u}{\partial p}=K^{-1}\left(p\right)\left[\frac{\partial f}{\partial p}-\left(\frac{\partial K}{\partial p}\right)u\right],$$

or in the index notation

$${\frac{\partial u}{\partial p}}_{i}=K^{-1}\left(p\right)\left[{\frac{\partial f}{\partial p}}_{i}-\left({\frac{\partial K}{\partial p}}_{i}\right)u\right].$$

Substituting $\frac{\partial u}{\partial p}$ back into Eq. 3 yielded

$$\frac{d\Pi }{dp_{i}}={\frac{\partial \Pi }{\partial p}}_{i}+{\left(\frac{\partial \Pi }{\partial u}\right)}^{T}K^{-1}\left(p\right)\left[{\frac{\partial f}{\partial p}}_{i}-\left({\frac{\partial K}{\partial p}}_{i}\right)u\right].$$ (6)

The only problematic term was $\frac{\partial \Pi }{\partial u}$ because it required solving the forward elasticity problem each time a small change was made to our unknown parameter $p_{i}$ . To address this issue, we wrote

$$\lambda^{T}={\left(\frac{\partial \Pi }{\partial u}\right)}^{T}K^{-1}\left(p\right),$$

where λ was named the adjoint variable. Rearranging yielded

$$K^{T}\left(p\right)\lambda =\frac{\partial \Pi }{\partial u}$$

Note that $\frac{\partial \Pi }{\partial u}=u^{sim}-u^{exp}$ from the definition, and $K^{T}=K$ from the symmetry of stiffness matrix. Therefore, to solve the adjoint equation, we applied the difference between the simulation and experiment displacements to drive the forward elasticity problem. Having solved for λ , the gradient was written as

$$\frac{d\pi }{dp_{i}}={\frac{\partial \Pi }{\partial p}}_{i}-\lambda^{T}\;\left[\left({\frac{\partial K}{\partial p}}_{i}\right)u-{\frac{\partial f}{\partial p}}_{i}\right],$$ (7)

where λ acted as a Lagrange multiplier. For our simpler case with no regularization $\left({\frac{\partial \Pi }{\partial p}}_{i}=0\right)$ and the external load being independent of the unknown material properties $\left({\frac{\partial f}{\partial p}}_{i}=0\right)$ , the derivative of the objective functional was simplified to

$$\frac{d\pi }{dp_{i}}=-\lambda^{T}\;\left[\left({\frac{\partial K}{\partial p}}_{i}\right)u\right].$$ (8)

To calculate this simplified objective functional gradient, the FE problem was solved two times: one was the forward elasticity problem to obtain u, and the second one was to solve the adjoint set of equations to get λ . To obtain the term ${\frac{\partial K}{\partial p}}_{i},$ we slightly perturbed the material property $p_{i}$ and collected the assembled stiffness matrix by using the matrix generation procedure available in Abaqus.

#### Particle Swarm Optimization Algorithm

In this well-known algorithm (Kennedy and Eberhart, 1995) a random population of *nPop* particles was initially generated. Each particle was basically a point in our search space for the optimal material properties. For example, in the homo/iso/hyper case, the position of each particle was defined by its value of $C_{10},\;C_{01,}$ and $D_{1}\;$ randomly drawn from a specified range given in Table 1. The FE model for each particle was solved and the corresponding error was evaluated. In order to update the position of the particles toward the location of the global minima, the velocity of each particle was updated based on:

$$v=\left[w\times v\right]+\left[c_{1}\times rand\left(\right)\left(p_{best}-p_{present}\right)\right]+\left[c_{2}\times rand\left(\right)\left(global_{best}-p_{present}\right)\right].$$ (9)

This determined the direction along which the value of the material properties was to be changed (i.e., increased or decreased). In Eq. 9, w was a damping factor which reduced the momentum of the particles as they iteratively progressed towards finding the global optima (Kennedy and Eberhart, 1995); it started from 1.0 and was multiplied by a constant of 0.99 after each iteration. The parameters *c*_*1*_ and *c*_*2*_ controlled the local and global search weights, respectively. $p_{best}$ and $p_{present}$ referred to the best score (smallest error) of a given particle throughout the whole iterations passed thus far and the one within the current iteration, respectively. The parameter $global_{best}$ referred to the best score of the overall population. The particles position was then updated by adding the calculated velocity to the current position. FE simulations were then performed for the whole batch of particles in an iterative fashion until the optimization algorithm converged.

In implementing the PSO algorithm, it was important to impose proper upper and lower bounds on the velocity and position vectors of each particle to avoid local minima or particles getting stuck in the neighborhood of each other or worse yet, on the boundaries (Kennedy and Eberhart, 1995). Prior to updating the position vectors using Eq. 9, any element of the velocity vector that had values above vMax or below vMin were set to vMax or vMin , respectively. Then we checked for the position vectors: if a particle’s position was beyond the limits defined by the range [varMin, varMax] , we checked its velocity vector; if it was pointing outside the position bound (meaning adding the velocity to the position would have resulted in the particle position landing outside of its permitted range), velocity component was set to zero, and the particle position was set to the corresponding upper or lower bound. The parameters $c_{1}$ and $c_{2}$ in Eq. 9 were set to be 2.0 (Kennedy and Eberhart, 1995). The ranges for particle velocity ([vMin, vMax]) were set as [−5, 5] for the homo/iso/hyper and homo/aniso/hyper cases, and as [−40, 40] for the hetero/iso/linear-elastic case. The parameter *nPop* was set as 24, 48, and 1,000 for the homo/hyper/iso, homo/aniso/hyper, and hetero/iso/linear-elastic cases, respectively. These hyperparameters maintained a wide parameter value range, helped the algorithm converge better, and were tuned based on our preliminary sensitivity analysis studies.

## Results

### The Interpolated Deformation and Strain Measures of the Lung

**FIGURE 3:**
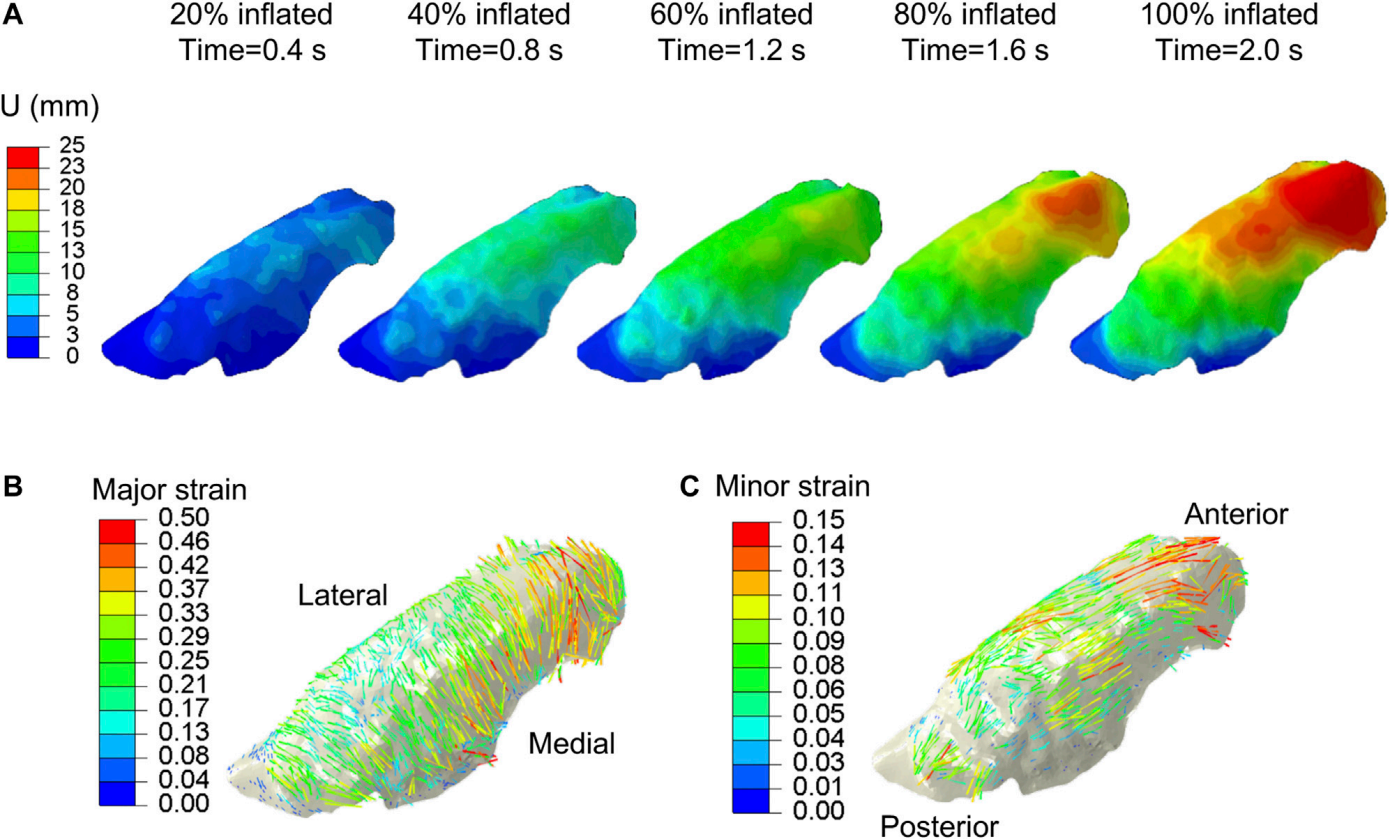
DIC measured displacements data, extracted at ∼7,000 evenly distributed points across the parenchymal surface were interpolated and applied to the FE model. **(A)** Displacement magnitude contour maps corresponding to five increments of 20% inflation steps. Anterior regions of the lung exhibited the largest distensions. Vector field map of major **(B)** and minor **(C)** strains were obtained from imposing interpolated DIC displacements to the model.

The experimental displacement values were imposed on the FE model using the generic homo/iso/hyper case to confirm the validity of the interpolation technique and the strain orientations against the DIC system calculations. The data matched nearly identically for each of the five inflation stages (0.4, 0.8, 1.2, 1.6, and 2.0 s) as shown in Figure 3, and was subsequently used in the optimization scheme. The motion of the lung during inspiration was substantially inhomogeneous: the anterior region of the lobe exhibited the greatest distention and pronounced aeration (as much as 25 mm). The imposed displacements at the nodes along the lobe perimeter were also non-zero and were interpolated to represent the actual boundary conditions of the deforming surface properly. The maximum and minimum in-plane principal strains (major and minor strains) obtained at the full inflation stage were shown in Figure 3B and Figure 3C, valuing no more than 0.5 and 0.15, respectively. The major strain predominantly aligned with the medial-lateral direction while the minor strain was preferentially aligned with the anterior-posterior direction.

### The Optimized Compound Material Properties of the Lung

The set of optimal material properties for the homo/iso/hyper and homo/aniso/hyper cases, and for a wide array of starting points, was listed in Tables 1, 2, respectively. The three material properties’ average ±standard deviation for the homo/iso/hyper was $C_{10}=136.5\pm 0.25$ kPa, $C_{01}=1.0\pm 0.0$ kPa, and $D_{1}=\left(13.43\pm 0.16\right)\times 10^{-4}$ kPa^−1^. The calculated shear and bulk moduli were $\mu_{0}=2\left(C_{10}+C_{01}\right)=275$ kPa, and $K_{0}=\frac{2}{D_{1}}=1.5$ MPa, respectively. The parameter $C_{01}$ , which controlled the contribution of ${\bar{I}}_{2}$ , consistently converged to its allowed lower bound of 1.0 kPa and was two order of magnitudes smaller than $C_{10}$ . The smallness of C01 in comparison to C10 indicates that the strain energy function is largely controlled by the stretch response of the tissue and not the distortion part. In ventilating the lung, the DIC strains also suggested that shear strains were minimal. Another consequence of C01 being very small is that the stress-strain curve would start to look less nonlinear.

The optimal set of material properties calculated for the homo/aniso/hyper case for seven optimization runs was $C_{10}=116.4\pm 0.2$ kPa, $D=\left(43.6\pm 29.2\right)\times 10^{-4}$ kPa^−1^, $k_{1}=1.0\pm 0.0$ kPa, $k_{2}=0.12\pm 0.06$ , and κ=0.33±0.0 (Table 2). The shear modulus was $\mu_{0}=2\left(C_{10}\right)=233$ kPa, comparable to the 275 kPa obtained for the previous homo/iso/hyper case.

While the three material parameters $C_{10}$ , $k_{1}$ , and κ converged to their optimal values, the model did not depend on D and $k_{2}$ . The anisotropic hyperelastic formulation in Abaqus ignores the compressibility coefficient and therefore, the objective function was simply insensitive to this parameter and *D* remained unchanged (Abaqus: Theory Manual, 2011). Conversely, *k*_*2*_, which was a dimensionless parameter exponentially controlling the contribution of the fibers in the overall strain energy function, did not yield a specific value because κ always converged to 0.33 and effectively zeroed out the anisotropic part of the strain energy density function. Therefore, $k_{2}$ , contributing to the anisotropic term, did not converge to a meaningful value because it played no role in the strain density function. Despite accounting for anisotropic lung behavior, the inverse optimization framework found no anisotropic advantage over the isotropic model. Given this observation, the homo/aniso/hyper case was not pursued further, and the considered models were limited to the homo/iso/hyper and hetero/iso/linear-elastic cases.

**FIGURE 4:**
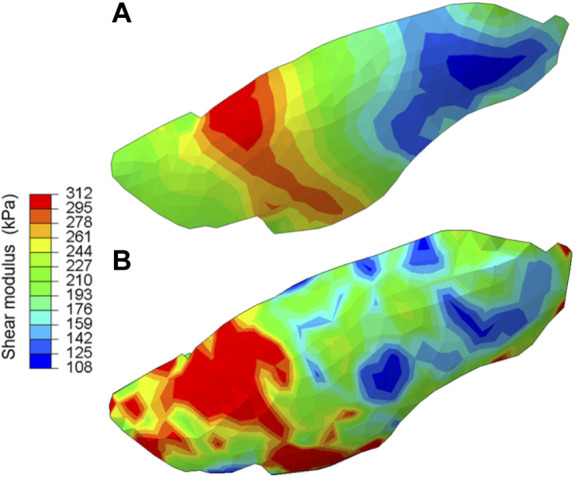
The shear modulus map obtained for the hetero/iso/linear-elastic case obtained from **(A)** the gradient-based and **(B)** derivative-free optimization algorithms. Both methods consistently demonstrated strong heterogeneity in the tissue elasticity distribution across the lung surface.

For the hetero/iso/linear-elastic case, we plotted the shear modulus map of the lung shown in Figure 4. In both SQP and PSO optimization schemes, the resulting spatial distribution of the shear modulus exhibited strong heterogeneity. The value for shear and bulk moduli was 108–312 kPa and 144 kPa–17.2 MPa, respectively, with the tissue softening from the posterior to anterior regions of the lobe.

**FIGURE 5:**
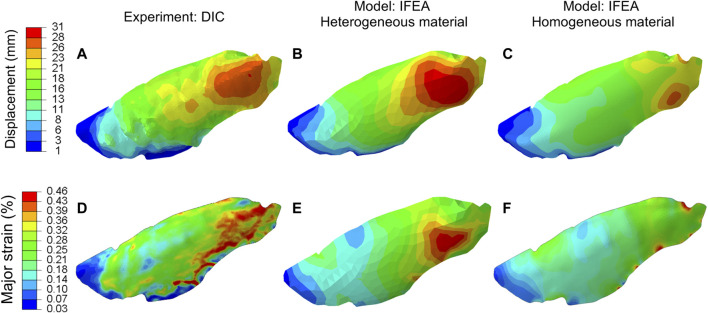
Comparison between the displacement **(A–C)** and major strain **(D–F)** of the DIC and IFEA with either homogeneous and heterogeneous material models. The computed displacement and strain contours of the heterogeneous model agreed with the DIC data better than the homogeneous model. The heterogeneous model results are based on the SQP algorithm, and the PSO algorithm yielded similar results.

The displacements and strains measured by DIC and predicted by the homogeneous and heterogeneous IFEA model were shown in Figure 5. The predictions of the heterogeneous model better matched the DIC displacement fields compared to that of the homogenous model. The overinflation of the lung at the anterior region was particularly well predicted by the heterogeneous model (Figure 5B).

**FIGURE 6:**
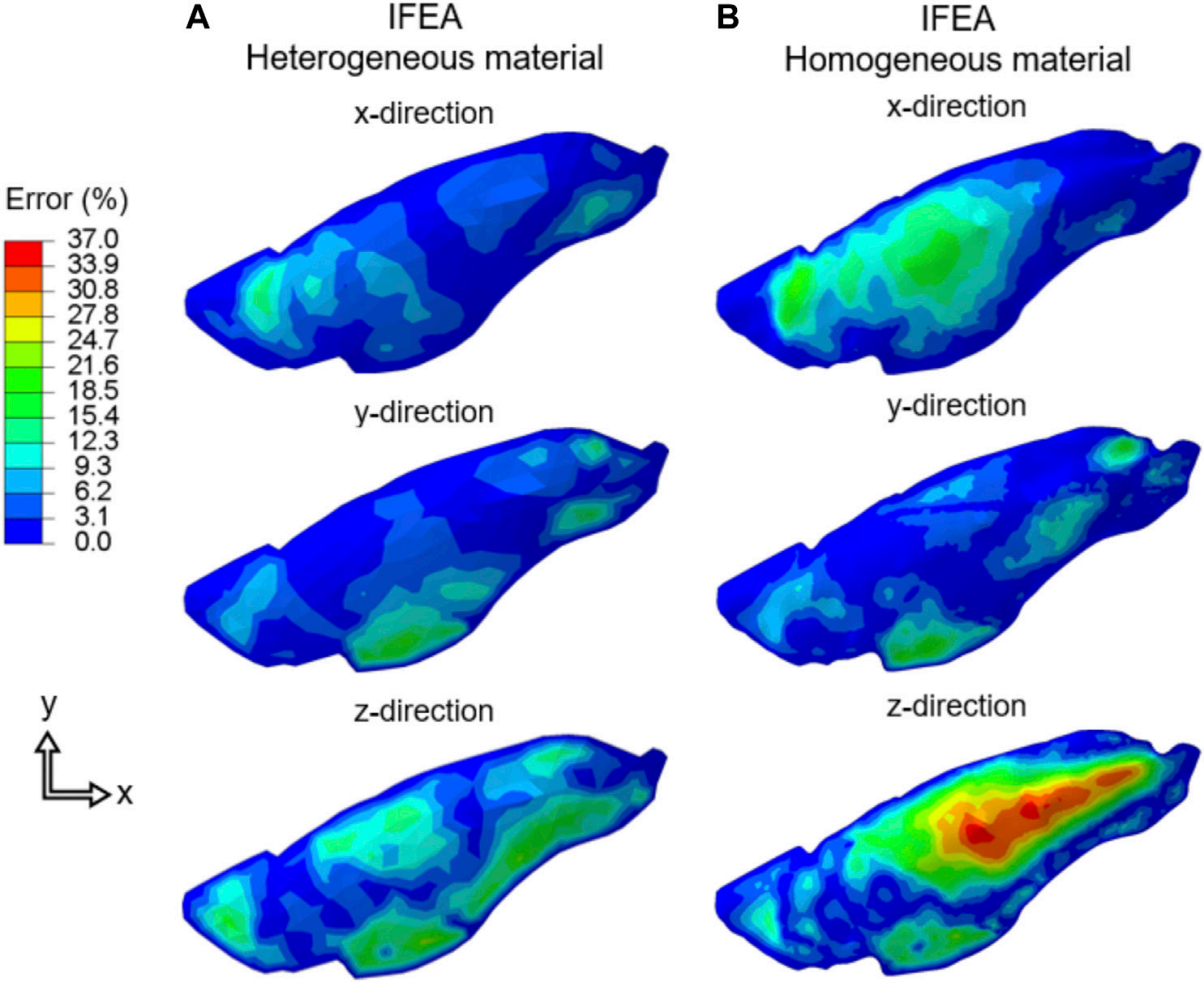
The displacement error (Cartesian components) of the IFEA predictions assuming **(A)** heterogeneous and **(B)** homogeneous material models. The errors for the heterogenous model were consistently smaller. SQP algorithm shown for the heterogeneous model error and the PSO algorithm yielded similar results.

The three components of displacement errors (percent normalized with respect of the maximum tissue displacement) for homogeneous and heterogeneous cases were shown in Figure 6. The errors for the heterogeneous case were consistently smaller than that of the homogeneous case and greatest in the *z*-direction (i.e., the direction at which DIC camera overlooked the lung), likely corresponding to the largest displacement values also being in the *z*-direction.

The overall IFEA settings and results of the two optimization algorithms applied to the homo/iso/hyper and hetero/iso/linear-elastic cases were summarized in Table 3. The homogeneous isotropic model returned an average error of 2.3 mm for both gradient-based and non-gradient-based optimization schemes. The average error for the heterogeneous lung model was consistently smaller than the homogeneous case; the PSO algorithm resulted in a slightly smaller error compared to the SQP algorithm (1.3 vs. 1.6 mm). The relative computational cost (CPU time normalized with respect to the fastest case) and number of iterations to reach the optimal solution were also given; the PSO algorithms for the homo/iso/hyper and hetero/iso/linear-elastic models were the fastest and most expensive simulations, respectively.

**TABLE 3:** General IFEA settings and results.

		**Homo/Iso/Hyper**	**Hetero/Iso/Linear-elastic**
Gradient-based optimization	Error (mm)	2.3	1.6
	Relative cost	2.7	3.1
	Algorithm	TRR	SQP
	Iterations	17	7
Non-gradient-based optimization	Error (mm)	2.3	1.3
	Relative cost	1.0	68.0
	Algorithm	PSO	PSO
	Iterations	30	77

## Discussion

While a one-to-one comparison between our reduced-order model and extracted pulmonary tissue specimen measures is impractical, we strived to compare the compound material parameters (averaging shear modulus of hundreds of kPa in all three constitutive models) with the reported ranges for parenchyma, airway, and pleura layer individual component responses (Tables 1, 2 and Figure 4). Our shear modulus values, representing the combined parenchyma, airway, and pleura layer materials, are greater than those of isolated lung parenchyma (0.17–0.27 kPa) and the alveolar wall (1.74 kPa), comparatively estimated by converting the previously reported elastic modulus with an assumed Poisson ratio of 0.43 (Lai-Fook et al., 1976; Cavalcante et al., 2005). Similarly converting the reported 10–70 kPa elastic moduli of isolated airway specimens (Eskandari et al., 2019) yields airway shear modulus range of 3.5–25 kPa, also less than our combined shear modulus results. Conversely, the encapsulating visceral pleura layer is approximated to have a shear modulus of ∼200 kPa at low stretch ratios (Humphrey et al., 1986), similar to our values.

The ratio between the bulk and shear modulus (often used to gage material compressibility) was nearly 5.5 for the homo/iso/hyper case, and 0.5 for the hetero/iso/linear-elastic case. This is significantly less than incompressible materials (with a ratio greater than 1,000) and justifies the use of the compressible material model, as previously suggested (Birzle and Wall, 2019). Our model suggests that lung elasticity is not distributed evenly across the regions, but that the shear moduli is smallest in the anterior region (Figure 4), corresponding to the location of maximum deformation. While literature substantiating this tissue heterogeneity across the organ is not yet available, regionally extracted tissue subjected to tensile or indentation tests can enable future comparisons.

Using this reduced-order model of the lung, our optimization scheme finds the anisotropic material model can be interchanged with an isotropic representation (since κ = 0.33) and still sufficiently capture the experimental displacements. While this finding is bound to the model limitation and calls for further experimental works to validate, it still can be substantiated given the isotropic material behavior of the parenchyma (Fung, 1988) where collagen and elastin fibers are randomly oriented (Toshima et al., 2004). However, the major strains were found to predominantly align with the medial-lateral direction while the minor strains were preferentially aligned with the anterior-posterior direction (Figure 3); this indicates that the spatial patterns and strain orientations were possibly a result of the geometry and loading of the lung more so than the anisotropic nature of the tissue material itself. An alternative hypothesis is the embedded monopodial main bronchial airway, which delivers oxygen from the anterior to posterior region and is twice as compliant circumferentially than axially, enables greater stretch in the medial-lateral direction (Sattari and Eskandari, 2020). Therefore, it is plausible that larger collagen-enriched airways may contribute to the anisotropic strain distribution in the lung to a great extent. Including a model mapping of the major airway pathways may help further differentiate the tissue matrix versus the effect of the structural reinforcement. This hypothesis will be explored further in upcoming mice and human lung experiments with differing bronchial branching patterns and collateral ventilation compared to the pig.

This reduced-order *in-silico* model of the lung facilitates a novel and much-needed class of inverse modeling approaches for the respiratory system. Current pulmonary biomechanical models of the lung can be categorized into two classes: 1) models primarily based on *in-vivo* kinematics data obtained from computed tomography (CT) or magnetic resonance (MR) images (Al-Mayah et al., 2010; Eom et al., 2010; Li et al., 2013; Ilegbusi et al., 2016; Ladjal et al., 2021), and 2) classic models idealizing the lung as single/multi resistive compartments calibrated with pressure-volume data (Bates, 2009). While these methods have been quite insightful, their shortcomings have motivated the novel approach put forward in this study. For instance, CT- and MR-based models (class 1) utilize convoluted and tedious deformable image registration (DIR), further challenged by the lack of a universal ground truth of lung nodules which necessitates expert-determined anatomical landmark detection and hinders model validation (Sotiras et al., 2013; Sarrut et al., 2017). Additionally, compartmentalized models (class 2) describe global bulk elastance and resistance behaviors and neglect the intricate multiscale architecture of the lung, omitting the local heterogeneities and strain risers responsible for inflammation and damage (Vlahakis et al., 1999; Gattinoni et al., 2003). The absence of controllable testing parameters and continuous measures *in-vivo* limits basic lung kinetic and kinematic investigations, such as exploring the role of ventilation volume and rate, and contrasting physiological negative-versus artificial positive-pressure ventilation (Eskandari et al., 2021). Our *ex-vivo* informed *in-silico* approach can readily establish the mechanical science of breathing by merging detailed data acquisition with computational predictions.

Forging a bridge between tissue-scale kinematics and organ-level kinetics facilitates fundamental explorations of multiscale characterization and inaugurates several applications. Generalizing and informing the model with multiple lung data sets and complete inhalation and exhalation breathing pressures can empower surgical planning strategies based on minimizing changes in strain patterns from lobectomies, segmentectomies, and wedge resections to preoperatively improve patient outcomes instead of postoperative evaluations (Charloux and Quoix, 2017). Extending this framework to include diseased lungs categories, such as fibrosis or asthma, will enable regional tissue remodeling detection studies to discover load- and deformation-based pathological deviations to guide therapies, as inspired by similar FE-based models of the lung being used to better aerosol deposition in asthmatic patients (Wall et al., 2010; Soni and Aliabadi, 2013) and optimal radiation therapy (Werner et al., 2009; Ilegbusi et al., 2016). Furthermore, this model has potential applications for the design of ventilators: many didactic (Anderson et al., 2009) or clinical ventilators (Zuckerberg et al., 2020) contain a lung-replicating rubber bladder or elastic balloon component with mechanical properties that are not physiologically representative. Our FE lung membrane-like model and optimized material properties can enhance the design resemblance of these ventilator systems to improve patient care.

This study has several limitations. Firstly, one animal was used to demonstrate this IFEA framework and therefore, statistically conclusive results regarding the material property values are not warranted. Second, while the optimization algorithms minimized the error, the final error was still not completely vanished; this indicates lung-specific constitutive models are needed to better replicate the DIC measurements, as concluded by earlier works (Eskandari et al., 2019). Third, while DIC allows continuous and evolutionary behaviors of the lung to be examined whereas digital volume correlation techniques are at discrete snapshots (Arora et al., 2021), DIC can only access the lung surface and the internal structure of the lung and the volumetric strain distributions are not represented; a potential enhancement to this technique could be the use of mirrors and prisms to collect multi-angled views. Fourth, the framework is built on *ex-vivo* setting, hence the predicted material properties are likely to be influenced by the deformation and kinetics of the ribcage and diaphragm. Fifth, the anisotropic formulation implemented in the FE-package used in this study does not take into account the compressibility aspect of plane-stress elements, such as membrane elements in our model and assumes constant volume instead while still allowing for reduction in the element thickness due to in-plane deformations. Thus, the compressibility coefficient (parameter D in the HGO model) is not optimized by the IFEA framework. New implementation of the HGO formulation that do indeed account for the compressibility are needed to further investigate the effects of this limitation and provide insight into the compressibility of the anisotropic model. Lastly, for simplicity and computational time considerations, the heterogeneous model was a linear model whereas a nonlinear model might result in a better calibrated model.

## Conclusion

This study establishes a computational model representing local lung kinetics by associating global organ-level pressures and volumes to tissue-level kinematics. This is achieved by developing a novel lung application IFEA framework informed and verified by *ex-vivo* continuous DIC measurements from a porcine lung controlled via a custom-designed respiration apparatus. The resulting FE model introduces a model constructed solely from the geometry and deformation of its external surface as a result of the applied inflation load. This *in-silico* reduced-order pulmonary surrogate consolidates complex lung tissues (i.e., the visceral pleura, bulk parenchymal tissue, and the airway tree) into a simplified 3-D surface model, yielding compound material properties of a membrane representative of pressure-deformation features of the lung. Furthermore, the heterogeneous lung elasticity map presented in this study empowers new avenues to improve characterization of diseased states by enabling region-specific assessments of mechanical remodeling, such as variations in tissue elasticity, thus far critically absent in the field.

## Data Availability

The raw data supporting the conclusion of this article will be made available by the authors, without undue reservation.
